# Computed tomography patterns predict clinical course of idiopathic pulmonary fibrosis

**DOI:** 10.1186/s12931-020-01562-2

**Published:** 2020-11-10

**Authors:** Byoung Soo Kwon, Jooae Choe, Kyung Hyun Do, Hee Sang Hwang, Eun Jin Chae, Jin Woo Song

**Affiliations:** 1 Division of Pulmonary and Critical Care Medicine, Department of Internal Medicine, Seoul National University Bundang Hospital, Seongnam-Si, Gyeonggi-Do South Korea; 2 Department of Radiology, University of Ulsan College of Medicine, Asan Medical Centre, Seoul, South Korea; 3 Department of Pathology, University of Ulsan College of Medicine, Asan Medical Centre, Seoul, South Korea; 4 Department of Pulmonology and Critical Care Medicine, University of Ulsan College of Medicine, Asan Medical Centre, 88 Olympic-ro 43-gil, Songpa-gu, Seoul, 05505 South Korea

**Keywords:** Idiopathic pulmonary fibrosis, Guideline, Survival, Respiratory function test

## Abstract

**Background:**

A new clinical guideline for idiopathic pulmonary fibrosis (IPF) uses high-resolution computed tomography (HRCT) patterns for diagnostic purposes. However, it is unknown how they relate to the IPF clinical course. We aimed to investigate whether HRCT patterns could be used to predict lung function changes and survival in patients with IPF.

**Methods:**

Clinical data were retrospectively reviewed in 337 patients with IPF (all biopsy-proven cases). HRCT patterns were classified according to the 2018 IPF diagnostic criteria.

**Results:**

The median follow-up was 46.9 months. The mean age was 62.5 years, and 74.2% were men. Among the HRCT patterns, usual interstitial pneumonia (UIP), probable UIP, indeterminate for UIP, and an alternative diagnosis were identified in 163 (48.4%), 110 (32.6%), 33 (9.8%), and 31 (9.2%) patients, respectively. The indeterminate for UIP group showed higher lung function and exercise capacity and better prognosis than the other groups. They also had a lesser decline in lung function than the other groups during follow-up. In the multivariate Cox analysis, which was adjusted by age, smoking status, lung function, exercise capacity, and use of antifibrotic agents, indeterminate for UIP pattern was found to be an independent prognostic factor (hazard ratio 0.559, 95% confidence interval 0.335–0.933, *P* = 0.026). However, the probable UIP group had similar lung function changes and prognosis when compared the UIP group.

**Conclusions:**

Our results suggest that indeterminate for UIP pattern on HRCT may predict a more favorable clinical course in patients with IPF, supporting the validity of the new IPF diagnostic guidelines.

## Introduction

Idiopathic pulmonary fibrosis (IPF) is the most common type of idiopathic interstitial pneumonia. It has the worst prognosis with a median survival time of 3 years [1]. The clinical course of IPF is variable [2], and predicting its prognosis is difficult. Previous studies have reported that baseline and change in lung function over time and exercise capacity are associated with poor prognosis in IPF [3–5]. Although high-resolution computed tomography (HRCT) is a pivotal modality for diagnosing IPF, it is unclear if it can be used for predicting the clinical course of IPF; previous studies on the association between HRCT patterns and survival, based on the 2011 IPF diagnostic criteria, showed inconsistent results [6, 7]. Salisbury et al. reported that biopsy-proven IPF patients with a possible usual interstitial pneumonia (UIP) pattern on HRCT had significantly prolonged survival than those with a definite UIP pattern in multivariate Cox analysis adjusted by age, gender, smoking status, pulmonary function, and fibrosis extent [6]. In contrast, Acardu et al. conducted a retrospective analysis of 350 patients with IPF and reported that a consistent UIP pattern was not associated with poorer survival than a possible or inconsistent pattern on HRCT when adjusted by age, sex, and lung function [7]. These contradictory findings may be due to differences in baseline demographic features and disease severity between studies. Therefore, the usefulness of HRCT in predicting clinical course of IPF is still unknown.

In 2018, a new clinical guideline for IPF was released, which reclassified the HRCT patterns into four categories: UIP, probable UIP, indeterminate for UIP, and alternative diagnosis [8]. The most important change from the previous guideline was that possible UIP pattern based on the 2011 guideline was subdivided into probable UIP and indeterminate for UIP [9]. Therefore, it can be assumed that a more detailed HRCT classification may be more useful in predicting the clinical course of IPF. However, it is unclear whether these newly defined HRCT patterns can be used to predict the clinical course of IPF. Therefore, the aim of this study was to evaluate the impact of HRCT patterns on clinical course including lung function changes and survival in patients with IPF.

## Material and methods

### Study population

**Fig. 1:**
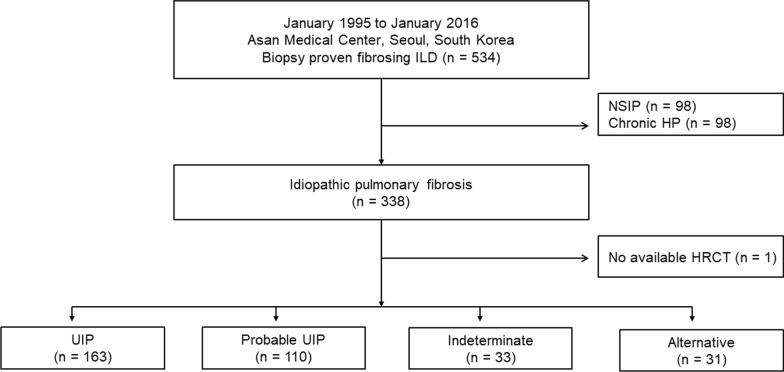
Patient disposition. IIP, idiopathic interstitial pneumonia; HP chronic hypersensitivity pneumonitis; NSIP, nonspecific interstitial pneumonia; HRCT, high resolution computed tomography; UIP, usual interstitial pneumonia

Between July 1995 and January 2016, 534 patients who underwent a surgical lung biopsy for diagnosing fibrosing interstitial lung disease were identified from the Asan Medical Centre, Seoul, South Korea. Among them, 196 patients were excluded due to other diagnoses, and one patient was excluded because of unavailability of HRCT images. Finally, 337 patients were included in this study (Fig. 1). All patients met the IPF diagnostic criteria of the 2018 American Thoracic Society (ATS), European Respiratory Society (ERS), Japanese Respiratory Society, and Latin American Thoracic Society statement. The diagnoses were confirmed through a multidisciplinary discussion. This study was approved by the Institutional Review Board of Asan Medical Centre (2018–1284), and informed consent was waived due to the retrospective nature of the study.

### Data collection

Clinical and survival data for all patients were retrospectively obtained from medical records, telephonic interviews, and/or the records of the National Health Insurance of Korea. The pulmonary function test results, forced vital capacity (FVC), total lung capacity (TLC), and diffusing capacity of the lung for carbon monoxide (DL_CO_) were measured based on the ATS/ERS recommendations [10–12]. The results are expressed as percentages of the normal predicted values. The six-minute walk test (6MWT) was performed according to the ERS/ATS recommendations [13].

Data from the follow-up visits (conducted every 3–6 months) or hospitalizations were reviewed to determine the development of complications, such as pneumonia, acute exacerbation (AE), lung cancer, and pneumothorax. AE was defined according to the criteria suggested by Collard et al. [14]

### HRCT evaluation

HRCT scans were obtained in accordance with standard protocols at full inspiration without contrast enhancement. The HRCT images acquired at the time of diagnosis were independently reviewed by three thoracic radiologists (JC, KHD, and EJC) blinded to the clinical and pathologic information. Overall, the HRCT pattern was categorized as UIP, probable UIP, indeterminate for UIP, and an alternative diagnosis, based on the IPF diagnostic criteria [8]. The UIP pattern was defined as subpleural, basal predominance of reticular abnormalities, honeycombing with or without traction bronchiectasis, and the absence of findings inconsistent with a UIP pattern including extensive ground-glass opacity, micronodules, discrete cysts, mosaic attenuation, or segmental/lobar consolidation [8]. Representative radiologic images of each HRCT pattern are shown in Additional file 1. Disagreement between the three readers was resolved via a consensus.

### Statistical analysis

All values are expressed as a mean ± standard deviation for continuous variables or as percentages for categorical variables. The Student’s t-test or the Mann–Whitney U test was used for continuous data, and Pearson’s chi-squared test or Fisher’s exact test was used for categorical data. The generalized interobserver agreement for all three readers was evaluated using Fleiss’ κ and an intraclass correlation coefficient (ICC). The strength of agreement was as follows: κ < 0.04 = poor agreement, 0.4–0.75 = good agreement, and κ > 0.75 = excellent agreement [15]. Survival was evaluated by the Kaplan–Meier survival analysis and the log-rank test. To estimate the survival time, we defined events as any cause of death or lung transplantation. The Cox regression model was used to identify the prognostic factors for survival. Variables with a *P*-value of < 0.1 in the univariate analysis were entered into the multivariate models (backward log-likelihood ratio statistics method). Lung function decline was estimated using a mixed-effect model with a random intercept and random slope, adjusting for age, sex, smoking history, and HRCT patterns as fixed effects and time as a random effect. All *P*-values were two-tailed. A *P*-value of < 0.05 was considered statistically significant. All statistical analyses were performed using SPSS version 22 (SPSS Inc., Armonk, NY, USA).

## Results

### Baseline characteristics

The median follow-up period was 46.9 months (interquartile range: 22.8–78.2 months). The mean age of the patients was 62.5 years; 74.2% were men, and 68.8% were ever-smokers. Among the HRCT patterns, the UIP pattern was the most common (48.4%), followed by the probable UIP pattern (32.6%), indeterminate for UIP pattern (9.8%), and alternative diagnosis (9.2%) (Fig. 1). The most common findings in the alternative diagnosis group were predominant ground-glass opacity, peribronchovascular distribution, and upper or mid-lung distribution (an additional file shows this in more detail [see Additional file 1: Table S1]). Interobserver agreement across the HRCT patterns showed moderate reliability (κ = 0.50, range 0.49–0.62; ICC = 0.81). Interobserver agreement according to the HRCT patterns was the highest in UIP pattern (κ = 0.62), followed by indeterminate for UIP pattern (κ = 0.47), probable UIP pattern (κ = 0.44), and alternative diagnosis (κ = 0.36).

There were significant differences in sex, smoking status, baseline lung function, and a saturation of oxygen during the 6MWT across four groups (Table 1). Patients with indeterminate for UIP pattern were more likely to be men and ever-smokers and had higher lung function (FVC, DLco, TLC) and oxygen saturation (initial and the lowest) during the 6MWT than the other groups. However, the 6MWT distance, baseline bronchoalveolar lavage fluid findings, and treatment during follow-up were not different among the four groups (Table 1).

**Table 1:** Comparison of the baseline characteristics in patients with IPF according to the HRCT patterns

	UIP	Probable	Indeterminate	Alternative	*P*-value
No. of patients	163	110	33	31	
Age, years	61.9 ± 7.6	62.9 ± 7.3	63.0 ± 7.7	63.5 ± 7.8	0.550
Male	133 (81.6)	71 (64.5)^†^	29 (87.9)	17 (54.8)^†^	< 0.001
Ever-smokers	126 (77.3)	66 (60.0) ^†^	28 (84.8)	12 (38.7) ^†^	0.001
PFT, % predicted (n = 336)	n = 163	n = 110	n = 33	n = 30	
FVC	69.9 ± 18.5^†^	73.7 ± 14.9^†^	84.6 ± 13.6	66.7 ± 18.5^†^	< 0.001
DL_CO_	57.6 ± 17.2^†^	66.5 ± 17.0	70.2 ± 21.1	58.4 ± 13.5^†^	< 0.001
TLC	69.7 ± 15.1^†^	73.1 ± 13.3^†^	83.5 ± 11.4	69.3 ± 15.3^†^	< 0.001
6MWT (n = 324)	n = 157	n = 107	n = 32	n = 28	
Distance, meter	448.2 ± 110.7	459.5 ± 95.4	475.3 ± 92.2	440.6 ± 110.5	0.461
Initial SpO_2_, %	96.0 ± 1.8^†^	96.5 ± 1.3^†^	97.3 ± 1.1	96.3 ± 1.7^†^	< 0.001
Lowest SpO_2_, %	89.5 ± 6.0^†^	92.2 ± 4.5	93.5 ± 5.0	89.7 ± 6.2^†^	< 0.001
BAL fluid analysis (n = 220)	n = 118	n = 72	n = 15	n = 15	
WBC, /μl	289.2 ± 278.4	305.0 ± 237.2	354.7 ± 305.5	233.5 ± 171.0	0.622
Neutrophil, %	12.0 ± 20.2	8.6 ± 15.3	6.5 ± 5.5	12.9 ± 18.8	0.438
Lymphocyte, %	13.2 ± 12.6	12.0 ± 10.4	15.9 ± 15.9	15.7 ± 13.9	0.570
Treatment					
Antifibrotic agents	56 (34.6)	46 (42.2)	12 (36.4)	5 (16.7)	0.258
Steroid ± IM	63 (38.9)	38 (34.9)	8 (24.2)	16 (53.3)	0.737
No treatment	43 (26.5)	25 (22.9)	13 (39.4)	9 (30.0)	0.392

Data are expressed as a mean ± standard deviation or a number (%) unless otherwise indicated

IPF, idiopathic pulmonary fibrosis; HRCT, high-resolution computed tomography; UIP, usual interstitial pneumonia; PFT, pulmonary function test; FVC, forced vital capacity; DL_CO_, diffusing capacity of the lung for carbon monoxide; TLC, total lung capacity; 6MWT, six-minute walk test; SpO_2_, saturation of peripheral oxygen; BAL, bronchoalveolar lavage; WBC, white blood cell; IM, immunosuppressant

^†^There was a statistically significant difference compared to indeterminate for UIP (*P* < 0.05)

### Lung function changes and complications

**Fig. 2:**
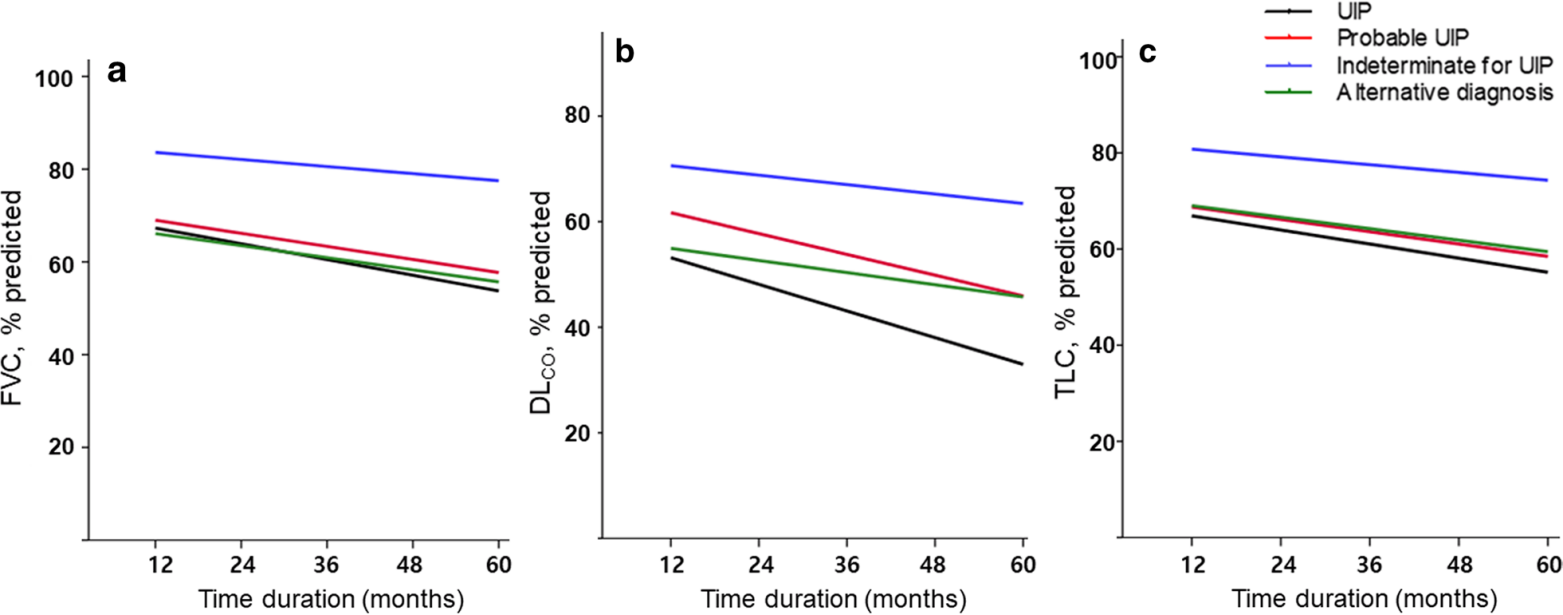
Lung function changes over 5 years in patients with IPF according to the HRCT patterns. These mixed-effect model plots were adjusted by age, sex, smoking history, and HRCT patterns as fixed effects and time as a random effect. Serial changes in **a** FVC (% predicted), **b** DL_CO_ (% predicted), and **c** TLC (% predicted) UIP, usual interstitial pneumonia; FVC, forced vital capacity; DL_CO_, diffusing capacity of the lung for carbon monoxide; TLC, total lung capacity

The indeterminate for UIP group displayed a significantly lesser decline rate of FVC (− 1.57% predicted/year) than the other groups (− 3.50% predicted/year in the UIP group, *P* < 0.001; − 2.79% predicted/year in the probable UIP group, *P* < 0.001; − 2.64% predicted/year in the alternative diagnosis group, *P* = 0.001) (Fig. 2a, an additional file shows this in more detail [see Additional file 1: Table S2]). In addition, the indeterminate for UIP group had a lesser decline rate of DLco (− 1.86% predicted/year) than the other groups (− 5.0% predicted/year in the UIP group, *P* < 0.001; − 3.98% predicted/year in the probable UIP group, *P* < 0.001) (Fig. 2b, an additional file shows this in more detail [see Additional file 1: Table S2]). Change in the TLC of the indeterminate UIP group (− 1.90% predicted/year) showed a lesser decline rate than that of the UIP (− 3.33% predicted/year in UIP, *P* < 0.001), probable UIP (− 2.57% predicted/year, *P* < 0.001), or alternative diagnosis group (− 2.38% predicted/year, *P* = 0.005) (Fig. 2c, an additional file shows this in more detail [see Additional file 1: Table S2]). During the follow-up period, a total of 137 (40.7%) patients were admitted to hospital due to complications. AE (56.9%) was the most common cause of hospital admission. There were no differences in the development of complications across the four groups. However, the indeterminate for UIP group showed a tendency of longer AE free survival period and a higher frequency of lung cancer than the other groups (Table 2).

**Table 2:** Comparison of the complications in patients with IPF according to the HRCT patterns

	UIP	Probable	Indeterminate	Alternative	*P*-value
No. of patients	163	110	33	31	
Unexpected respiratory hospitalization	66 (42.9)	49 (40.9)	9 (27.3)	13 (41.9)	0.393
Acute exacerbation	36 (22.1)	32 (29.1)	4 (12.1)	6 (19.4)	0.564
Time interval from diagnosis, months	36.7 ± 42.2^†^	46.6 ± 39.8^†^	92.9 ± 67.5	47.5 ± 26.2	0.089
Pneumonia	24 (14.7)	17 (15.5)	3 (9.1)	5 (16.1)	0.857
Pneumothorax	6 (3.7)	0 (0.0)	2 (6.1)	2 (6.5)	0.497
Lung cancer	24 (14.7)	8 (7.3)	5 (15.2)	1 (3.2)	0.092

Data are expressed as a mean ± standard deviation or as a number (%)

IPF, idiopathic pulmonary fibrosis; HRCT, high-resolution computed tomography; UIP, usual interstitial pneumonia

^†^There was a statistically significant difference compared to indeterminate for UIP (*P* < 0.05)

### Survival and prognostic factors

**Fig. 3:**
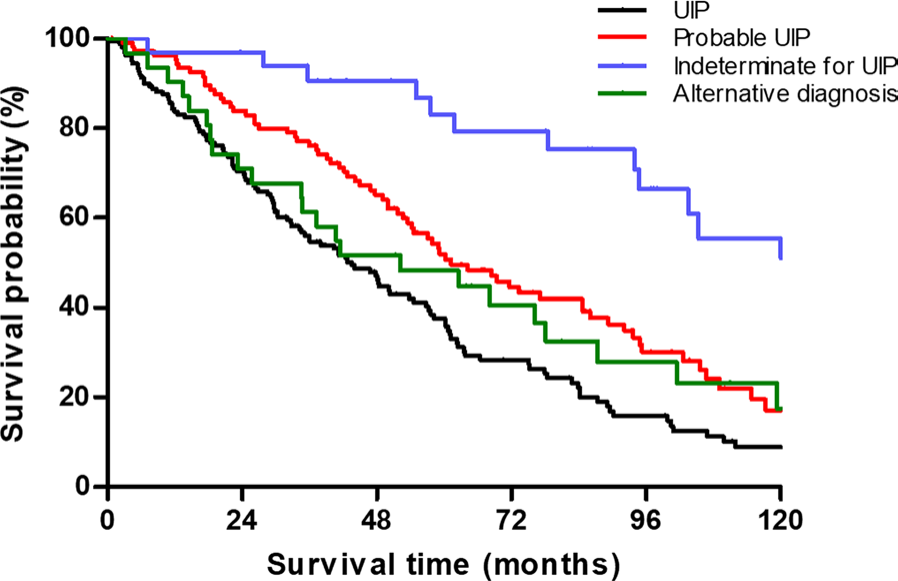
Comparison of the survival curves in patients with IPF according to the HRCT patterns. IPF, idiopathic pulmonary fibrosis; HRCT, high-resolution computed tomography; UIP, usual interstitial pneumonia

During follow-up, 70.0% of the total patients died, and 3.3% of patients underwent a lung transplantation. The median survival time of all subjects was 57.2 months. The indeterminate for UIP group displayed better survival (median survival period: 130.3 months) than the other groups (43.5 months in the UIP group, *P* < 0.001; 61.2 months in the probable UIP group, *P* = 0.005; 52.1 months in the alternative diagnosis group, *P* = 0.003) (Fig. 3).

In the univariate Cox analysis, probable UIP pattern and indeterminate for UIP pattern on the HRCT were significant predicting factors for survival in IPF patients along with younger age, ever-smoker, higher FVC, TLC, and DL_CO_, longer 6MWT distances, higher baseline, and the lowest oxygen saturation during the 6MWT, and anti-fibrotic treatment (Table 3). In the multivariate analysis, an indeterminate for UIP pattern on the HRCT was also an independent prognostic factor (hazard ratio [HR] 0.559, 95% confidence interval [CI] 0.335 – 0.933, *P* = 0.026) in IPF patients along with an alternative diagnosis pattern, younger age, higher FVC, longer walking distance and higher nadir oxygen saturation during the 6MWT, and antifibrotic treatment (Table 3).

**Table 3:** Prognostic factors for the mortality in patients with IPF assessed using Cox’s proportional hazards model

	Univariate		Multivariate	
	Hazard ratio (95% CI)	*P*-value	Hazard ratio (95% CI)	*P*-value
Age	1.035 (1.016–1.054)	< 0.001	1.042 (1.021–1.064)	< 0.001
Male	0.879 (0.647–1.196)	0.413		
Ever-smokers	0.727 (0.554–0.954)	0.021		
FVC	0.963 (0.955–0.972)	< 0.001	0.974 (0.963–0.985)	< 0.001
DL_CO_	0.968 (0.960–0.976)	< 0.001		
TLC^†^	0.957 (0.947–0.967)	< 0.001		
6MWT, distance	0.996 (0.994–0.997)	< 0.001	0.998 (0.997–1.000)	0.049
6MWT, initial SpO_2_	0.794 (0.734–0.859)	< 0.001		
6MWT, lowest SpO_2_	0.910 (0.893–0.922)	< 0.001	0.948 (0.923–0.974)	< 0.001
Antifibrotic agents	0.392 (0.290–0.530)	< 0.001	0.446 (0.324–0.615)	< 0.001
HRCT pattern		< 0.001		0.002
UIP	1		1	
Probable UIP	0.598 (0.446–0.802)	0.001	0.781 (0.565–1.078)	0.132
Indeterminate	0.299 (0.179–0.499)	< 0.001	0.431 (0.254–0.732)	0.002
Alternative	0.725 (0.467–1.124)	0.150	0.520 (0.318–0.849)	0.009

IPF, idiopathic pulmonary fibrosis; CI, confidence interval; PFT, pulmonary function test; FVC, forced vital capacity; DL_CO_, diffusing capacity of the lung for carbon monoxide; 6MWT, six-minute walk test; SpO_2_, saturation of peripheral oxygen; HRCT, high-resolution computed tomography; UIP, usual interstitial pneumonia

^†^TLC was excluded from the multivariate analysis due to its close correlation with FVC

## Discussion

In this study, IPF patients with an indeterminate for UIP pattern, which was a newly defined category in the revised IPF diagnostic criteria, exhibited more preserved lung function and a better clinical course (lesser decline in lung function and higher survival rate) than those with other HRCT patterns. Even when adjusted by clinical variables, including the baseline lung function and antifibrotic treatment, an indeterminate for UIP pattern independently predicted better survival. Conversely, IPF patients with probable UIP pattern had similar prognosis when compared with those with UIP pattern.

Chung and colleagues, in 201 patients with pulmonary fibrosis who had lung tissue samples taken, suggested that the possible UIP pattern could be subdivided into probable and indeterminate for UIP [16]. Indeterminate for UIP is defined as having subtle reticulation or ground-glass opacity on HRCT that is not classifiable into the other categories [8]. They demonstrated that histologic UIP was proven in 82.4% of patients with probable UIP on HRCT and 54.2% of patients with indeterminate UIP (*P* = 0.01). In our study using biopsy-proven IPF patients, the indeterminate for UIP group had a better prognosis than the other groups. These findings implicated that the HRCT patterns may predict different clinical courses, as well as histologic correlation. Although it might be thought that a favorable prognosis is related to an early diagnosis, the findings of our study, in which the indeterminate for UIP group has been identified as an independent prognostic factor even when adjusted by age, lung function, exercise capacity, and antifibrotic treatment, suggest that the clinical course of the indeterminate group might be different from that of the other groups.

In our study, there were no significant differences in survival between IPF patients with the UIP pattern on HRCT and those with the probable UIP pattern. To date, inconsistent results regarding the association between survival and HRCT patterns were shown [6, 7, 17]. Arcadu et al. in 350 patients with IPF (197 biopsy-proven cases), showed no significant difference in survival between patients with a UIP pattern and those with a non UIP pattern (possible and/or inconsistent UIP pattern; HR 1.29, 95% CI 0.90–1.83, *P* = 0.15) on HRCT [7]. Lee et al. in 606 patients with IIP whose HRCT pattern was UIP (n = 544) or possible UIP (n = 62), showed that a 3-year survival rate was different according to the HRCT patterns (44.6% in the UIP group vs. 56.8% in the possible UIP group, *P* = 0.04). However, a propensity matching analysis of 122 patients (61 in the UIP group and 61 in the possible UIP group) showed that there was no survival difference between the two groups (48.7% vs. 61.1%, *P* = 0.17) [17]. In contrast, Salisbury et al. in 133 patients with biopsy-proven IPF, reported that patients with a definite UIP pattern on HRCT (n = 41) had a shorter survival period than those with a possible UIP pattern (median survival period: 2.27 vs. 6.95 years, *P* = 0.002) [6]. These conflicting results may be due to heterogeneous populations (various diagnoses and disease severity) in each study. Recently, Fukihara et al. in 311 IPF patients with HRCT pattern of UIP (n = 154) or probable UIP (n = 157), reported that the HRCT patterns were not significantly associated with survival in the multivariate analysis adjusted by age, sex, FVC, DL_CO_, and use of antifibrotic agents (HR 0.883, 95% CI 0.640–1.218, *P* = 0.447) [18]. They categorized the HRCT patterns according to the revised diagnostic criteria, and showed consistent results with our findings. Therefore, insignificant survival differences between patients with UIP and probable UIP pattern on HRCT may advocate the current guidelines, in which surgical lung biopsy is conditionally recommended in the probable UIP pattern [8, 19].

There were significant differences in lung function decline in IPF patients according to the HRCT patterns in our study. Although lung function changes were not significant between the UIP and the probable UIP group, lesser lung function changes were notable in the indeterminate for UIP group. Similarly, Raghu et al. in a post hoc subgroup analysis from the INPULSIS trial, reported that among the placebo group, the rate of lung function decline in patients who had a possible UIP pattern with traction bronchiectasis on HRCT was comparable to that in patients with a UIP pattern on a surgical lung biopsy or HRCT (− 221.0 vs. − 225.7 ml/year) [20]. However, any other data have not existed on the trajectory of lung function according to each HRCT pattern. Instead, studies on the association between lung function changes and disease severity stratified into baseline FVC were reported [21, 22]. Helen et al. in 416 patients with IPF, showed that the annual decline in FVC was not different (− 4.6% predicted/year vs. − 4.9% predicted/year, *P* = 0.779) between patients with FVC ≥ 80% predicted and those with FVC < 80% predicted [21]. Furthermore, Kolb et al. using the placebo group from the INPULSIS trial, showed that the rate of decline in FVC per year in IPF patients with baseline FVC ≥ 90% predicted was similar to that in those with FVC < 90% predicted (− 224.6 ml/year vs. − 223.6 ml/year) [22]. Considering that patients with an indeterminate for UIP pattern showed better lung function (mean FVC ≥ 80% predicted) than the other groups, our results were inconsistent with previous findings. These contradictory findings may be due to differences in treatment status of the study population (no treatment [22] or a small number of patients receiving antifibrotic agents [21]). Recently, Cocconcelli et al. in 49 patients with IPF naïve of antifibrotic agents, showed that changes in alveolar score, defined as extent of ground-glass opacities, were associated with FVC decline (r = 0.66, *P* = 0.002) [23]. Although our study did not use a quantitative scoring system, our results suggest that along with quantitative measure and lung function, qualitative evaluation of HRCT at baseline could be a useful predicting prognosis.

In the current study, lung cancer tended to be more likely to be developed in patients with a UIP pattern on HRCT in addition to those with indeterminate for UIP pattern. However, previous study showed results inconsistent with our findings; Almeida et al. in 244 patients with ILD, reported that there were no significant differences in the lung cancer incidence according to the HRCT pattern (8.5% in UIP, 10.5% in probable UIP, and 16.7% in indeterminate UIP, *P* = 0.551) [24]. However, of the all subjects, only 38.4% had IPF and 29.1% was ever-smokers. In patients with IPF, an exceedingly high proportion of carcinomas, including squamous carcinomas, develop at the same sites where fibrosis and tissue remodelling are predominant (peripheral and/or basal lung areas), and are topographically associated with honeycomb lesions and epithelial metaplasia [25, 26]. Calio et al. also demonstrated that the immunohistochemical characterization of lung cancer in IPF patients exhibited more varying bronchiole-related markers than that in non-IPF patients, [27] suggesting that lung cancer in IPF arise from transformed small airways in honeycomb lung areas where abnormal bronchiolar proliferation takes place. These results support our finding showing higher incidences of lung cancer in IPF patients with an UIP pattern on HRCT.

This study has several limitations. First, this study was conducted in a single centre and had a retrospective design. However, the baseline characteristics of our patients were similar to those of previous studies [6, 17, 20]. Second, our study only included IPF patients confirmed by surgical lung biopsy. As surgical lung biopsies cannot be done in patients that have a high risk of postoperative complications, the included subjects may have better-preserved lung function. Nevertheless, when lung function was adjusted in the multivariate Cox analysis, the HRCT pattern was still a significant predicting factor for survival in patients with IPF. Third, the number of patients with indeterminate for UIP pattern or alternative diagnosis, compared to that with UIP or probable UIP pattern on HRCT, was small. Previous studies reported that 35–60% of interstitial lung disease patients with a pathologic UIP pattern showed an inconsistent UIP pattern on HRCT [16, 28]. However, the current study included only those diagnosed with IPF through a surgical lung biopsy, excluding nonspecific interstitial pneumonia or chronic hypersensitivity pneumonitis. Even considering a small number of subjects, our results suggest the usefulness of HRCT in predicting survival in patients with IPF. Finally, interobserver agreement for HRCT patterns was not high. However, interobserver agreement in our study is comparable to that of previous study (kappa value 0.66–0.69) [29]. A recent study of 98 patients with ILD showed that the new software, IPFdatabase, can improve interobserver agreement among radiologists (kappa value of 0.18 to 0.64) [30]. Further studies on enhancing the interobserver agreement are warranted.

In conclusion, an indeterminate for UIP pattern on HRCT was associated with more stable lung function changes and better survival in patients with IPF. These results suggest that HRCT may be useful in classifying subgroups with a better prognosis among patients with IPF. This supports the validity of the new classification guidelines regarding HRCT patterns.

## Supplementary information

**Additional file 1: Table S1.** HRCT findings inconsistent with UIP pattern in IPF patients with an alternative diagnosis on HRCT. **Table S2.** Comparison of lung function changes in patients with IPF according to the HRCT patterns. **Figure S1.** Representative radiologic images of each group.

## Data Availability

The datasets used and/or analysed during the current study are available from the corresponding author on reasonable request.

## Abbreviations

DL_CO_: Diffusing capacity of the lung for carbon monoxide
FVC: Forced vital capacity
HRCT: High-resolution computed tomography
IPF: Idiopathic pulmonary fibrosis
PFT: Pulmonary function test
TLC: Total lung capacity
UIP: Usual interstitial pneumonia
6MWT: Six-minute walk test
