# Supplementary material for: Computed tomography patterns predict clinical course of idiopathic pulmonary fibrosis
Source: Respir Res. 2020 Nov 10;21:295. doi: 10.1186/s12931-020-01562-2 (PMC7653759; doi:10.1186/s12931-020-01562-2)

**Additional file 1**

**Computed tomography patterns predict clinical course of idiopathic pulmonary fibrosis**

Byoung Soo Kwon^1^, Jooae Choe^2^, Kyung Hyun Do^2^, Hee Sang Hwang^3^, Eun Jin Chae^2^, Jin Woo Song^4^

^1^Division of Pulmonary and Critical Care Medicine, Department of Internal Medicine, Seoul National University Bundang Hospital, Seongnam-Si, Gyeonggi-Do, South Korea

^2^Department of Radiology, University of Ulsan College of Medicine, Asan Medical Center, Seoul, South Korea.

^3^Department of Pathology, University of Ulsan College of Medicine, Asan Medical Center, Seoul, South Korea.

^4^Department of Pulmonology and Critical Care Medicine, University of Ulsan College of Medicine, Asan Medical Center, Seoul, South Korea.

**Table S1.** **HRCT findings inconsistent with UIP pattern in IPF patients with an alternative diagnosis on HRCT**

| HRCT findings |  |
| --- | --- |
| Patients number | 31 (100.0) |
| HRCT features |  |
| Cysts | 0 (0.0) |
| Marked mosaic attenuation | 4 (12.9) |
| Predominant GGO | 6 (19.4) |
| Profuse micronodules | 1 (3.2) |
| Centrilobular nodules | 1 (3.2) |
| Nodules | 1 (3.2) |
| Consolidation | 3 (9.7) |
| Predominant distribution |  |
| Peribronchovascular distribution | 6 (19.4) |
| Perilymphatic | 0 (0.0) |
| Upper or mid-lung | 6 (19.4) |
| Other |  |
| Pleural plaques | 0 (0.0) |
| Dilated esophagus | 0 (0.0) |
| Distal clavicular erosion | 0 (0.0) |
| Extensive lymph node enlargement | 0 (0.0) |
| Pleural effusion | 1 (3.2) |
| Pleural thickening | 0 (0.0) |

Data are expressed as a number (%).

UIP, usual interstitial pneumonia; IPF, idiopathic pulmonary fibrosis; HRCT, high-resolution computed tomography; GGO, ground-glass opacity

**Table S2. Comparison of lung function changes in patients with IPF according to the HRCT patterns**

|  | **Estimate** | **95% CI** | ***P-*value** |
| --- | --- | --- | --- |
| FVC % predicted |  |  |  |
| UIP | -0.515 | -0.649 – -0.380 | <0.001 |
| Probable UIP | -0.361 | -0.498 – -0.224 | <0.001 |
| Alternative | -0.313 | -0.499 – -0.127 | 0.001 |
| Indeterminate | Reference |  |  |
| DL_CO_ % predicted |  |  |  |
| UIP | -0.896 | -1.067 – -0.726 | <0.001 |
| Probable UIP | -0.565 | -0.739 – -0.391 | <0.001 |
| Alternative | -0.204 | -0.440 – 0.031 | 0.089 |
| Indeterminate | Reference |  |  |
| TLC % predicted |  |  |  |
| UIP | -0.370 | -0.481 – -0.258 | <0.001 |
| Probable UIP | -0.268 | -0.382 – -0.155 | <0.001 |
| Alternative | -0.220 | -0.373 – -0.067 | 0.005 |
| Indeterminate | Reference |  |  |

IPF, idiopathic pulmonary fibrosis; HRCT, high-resolution computed tomography; UIP, usual interstitial pneumonia; FVC, forced vital capacity; DL_CO_, diffusing capacity of the lung for carbon monoxide; TLC, total lung capacity; CI, confidence interval

Figure S1. Representative radiologic images of each group

(A) UIP, (B) Probable UIP, (C) Indeterminate for UIP, and (D) Alternative diagnsosis. UIP, Usual interstitial pneumonia


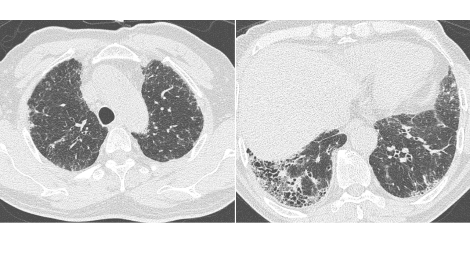


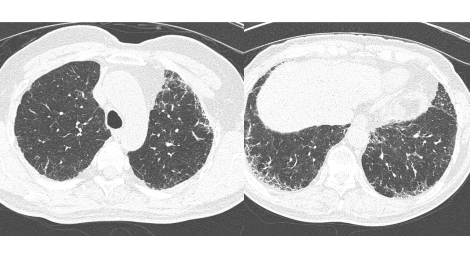


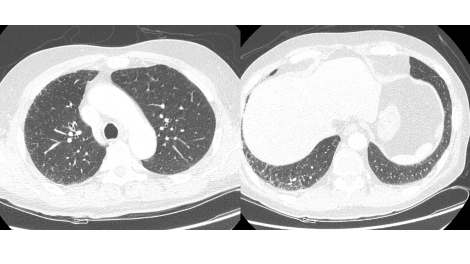


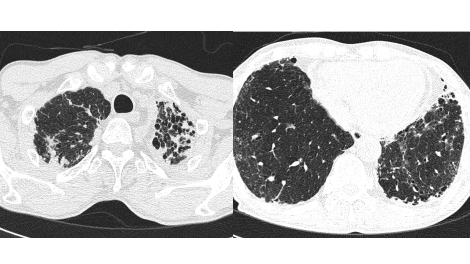

Supplement: Supplementary file 1 — Additional file 1: Table S1. HRCT findings inconsistent with UIP pattern in IPF patients with an alternative diagnosis on HRCT. Table S2. Comparison of lung function changes in patients with IPF according to the HRCT patterns. Figure S1. Representative radiologic images of each group. [file 12931_2020_1562_MOESM1_ESM.docx]
